# Psychiatric adverse events associated with semaglutide, liraglutide and tirzepatide: a pharmacovigilance analysis of individual case safety reports submitted to the EudraVigilance database

**DOI:** 10.1007/s11096-023-01694-7

**Published:** 2024-01-24

**Authors:** Mansour Tobaiqy, Hajer Elkout

**Affiliations:** 1 Department of Pharmacology, College of Medicine, University of Jeddah, Jeddah, Saudi Arabia; 2 Department of Family and Community Medicine, Medical Faculty, University of Tripoli, Tripoli, 13275 Libya

**Keywords:** Liraglutide, Obesity, Psychiatric adverse events, Semaglutide, Suicide, Tirzepatide

## Abstract

**Background:**

Semaglutide, liraglutide and tirzepatide are glucagon-like peptide-1 (GLP-1) receptor agonists that are effective for weight reduction. Recent reports of patients experiencing suicidal thoughts and other psychiatric adverse events while using GLP-1 agonists have raised concerns about the potential risk of self-harm and led the European Medicines Agency to investigate these medications.

**Aim:**

To identify and analyse the psychiatric adverse events associated with semaglutide, liraglutide and tirzepatide.

**Method:**

All individual case safety reports for semaglutide, liraglutide, and tirzepatide reported to the EudraVigilance database from 01/01/2021 to 30/05/2023 were analysed. Descriptive statistics were used to explore study population characteristics.

**Results:**

During the study period, 31,444 adverse event reports were identified: semaglutide (n = 13,956; 44.4%), liraglutide (n = 16,748; 53.2%), and tirzepatide (n = 740; 2.3%). There were 372 reports with psychiatric adverse event reports (n = 372; 1.18%) with a total of 481 adverse events. Women accounted for 65% (n = 242) of these reports. Depression was the most commonly reported adverse event (n = 187; 50.3%), followed by anxiety (n = 144; 38.7%) and suicidal ideation (n = 73; 19.6%). Nine deaths (8 with liraglutide and 1 with semaglutide) and 11 life-threatening outcomes (4 associated with liraglutide and 7 with semaglutide) were reported. The fatal outcomes occurred primarily among men (8 out of 9) resulting from completed suicidal attempts and depression.

**Conclusion:**

Psychiatric adverse events comprised only 1.2% of the total reports for semaglutide, liraglutide, and tirzepatide. However, the severity and fatal outcomes of some of these reports warrant further investigation.

**Supplementary Information:**

The online version contains supplementary material available at 10.1007/s11096-023-01694-7.

## Impact statements

- Healthcare professionals should be aware of the potential for psychiatric adverse events, particularly depression, anxiety and suicidal ideation associated with semaglutide, liraglutide, and tirzepatide. Patients should be encouraged to report any changes in mood or behavior to their healthcare provider and drug safety authority.
- Patients starting treatment with these medications should be screened for pre-existing psychiatric conditions and monitored regularly for signs and symptoms of depression, anxiety, and suicidal ideation.
- Regulatory agencies should carefully review the safety data for GLP-1 receptor agonists and consider requiring additional warnings and precautions on drug labels. The ongoing monitoring of post-marketing surveillance data is essential to identify any emerging safety concerns.
- Neuropsychiatric safety should be a key consideration in any randomized clinical trial and clinical risk-benefit assessment associated with GLP1 anti-obesity medications and the data provided here contribute to thoughtful and objective decision-making and dialogue between patients and clinicians.

## Introduction

Obesity is a global health challenge, with few pharmacologic options available for treatment. Weight reduction is undoubtedly essential for improving the outcomes for people with obesity and type 2 diabetes mellitus (T2DM) [1]. The Glucagon-like peptide-1 receptor agonists (GLP-1RAs) are a class of medicines that mimic the effects of GLP-1 to regulate blood sugar levels, including stimulating insulin secretion, suppressing glucagon secretion, and slowing gastric emptying. They bind to the GLP-1 receptor on cells in the pancreas and gut and are used to treat T2DM and obesity [2–5].

Semaglutide, liraglutide, and tirzepatide are injectable GLP-1RA drugs that act as GLP-1 receptor agonists [6–9]. However, tirzepatide is a novel dual glucose-dependent insulinotropic polypeptide (GIP) and GLP-1 receptor agonist that combines the actions of both incretin hormones into a single molecule [7]. All three drugs exhibit significant weight loss effects in patients with obesity or who are overweight with comorbidities in various clinical trials. Tirzepatide has superior weight loss potential compared with semaglutide or liraglutide [6–9].

The FDA (United States Food and Drugs Administration) first approved liraglutide in 2010 for the treatment of T2DM. In 2014, it was also approved for the treatment of obesity, whereas tirzepatide was first approved by the FDA in 2022 for the treatment of T2DM. It is also currently being studied for the treatment of obesity [10]. Semaglutide at 2.4 mg once weekly plus lifestyle intervention was associated with clinically meaningful weight loss in adults with obesity [11].

A systematic review was conducted for subcutaneous semaglutide, which involved six placebo-controlled and seven active-controlled trials. Semaglutide significantly decreased glycated hemoglobin (A1C) levels compared with sitagliptin, liraglutide, exenatide ER and dulaglutide but adverse reactions (nausea, vomiting) were more likely to occur with semaglutide [12].

Clinical trials of GLP-1 anti-diabetic and anti-obesity medications have reported varied adverse drug reactions (ADRs). According to Marso et al. (2016), the most common ADRs associated with liraglutide were mild or moderate nausea and diarrhea. Serious adverse events occurred in 6.2% of patients in the liraglutide group and 5.0% in the placebo group. Thirteen patients had pancreatic cancer in the liraglutide group and 5 in the placebo group but the finding was not significant. Gastrointestinal (GIT) adverse events were the most common, leading to the discontinuation of liraglutide. However, no psychiatric ADRs associated with liraglutide were reported in the trial [13].

In a trial involving tirzepatide and semaglutide, GIT adverse events such as nausea, vomiting, and diarrhea were the most commonly reported. The incidence ranged between 6 to 22%. However, no psychiatric adverse events (AES) were reported in this trial [14]. A similar safety profile was observed in the SURMOUNT-2 randomized trial of tirzepatide in adults with obesity and T2DM where GIT adverse events were the most frequently reported with no data on neuro or psychiatric adverse events available [15].

Common adverse effects of GLP-1 receptor agonists include nausea, vomiting, diarrhea, constipation, abdominal pain, dyspepsia, headache and nasopharyngitis [16]. Although there is a concern for the development of rare pancreatitis, pancreatic cancer or thyroid cancer, a meta-analysis of several databases did not suggest any increased risk of acute pancreatitis or pancreatic cancer with GLP-1RA treatment in T2DM patients [17].

Neuropsychiatric safety issues have become an area of interest given that many approved anti-obesity medications are centrally acting appetite suppressants [18, 19]. Rimonabant, the first selective central cannabinoid (CB1) receptor antagonist, was not approved by the FDA because of concerns regarding psychiatric adverse events including depression, anxiety and suicidal ideation [20]. Rimonabant was authorized in Europe in 2006 as an anti-obesity medication and then withdrawn by the European Medicines Agency (EMA) in 2009 leading to the termination of the development of other cannabinoid receptor 1 (CB1) blockers for obesity [21, 22].

Depression is a prevalent mood disorder and a significant health concern that often co-occurs with other diseases including metabolic disorders like T2DM and obesity [23]. Studies have shown that people living with obesity and T2DM are more likely to experience mental health issues [23]. Treatment with GLP-1RAs may be helpful for the treatment of depressive disorders [24]. Some studies have indicated that administering GLP-1RAs may directly exert an antidepressant effect on experiments with animals. [24].

A retrospective US FDA Adverse Event Reporting System (FAERS) analysis from January 2013 to June 2020 reported 18,675 unique AEs associated with anti-obesity medications involving 15,143 patients. The average patient age was 49.8 years with a majority of female adults (73.4%). The most frequent AEs are nausea, vomiting, dizziness, headache, drug ineffectiveness, cardiovascular issues and kidney complications. Adverse events outcomes (n = 21,229) were reported including 1,039 deaths (4.9% of all reports), 1,613 life-threatening events (7.6%), and 7,426 hospitalizations (35%). Phentermine/topiramate is associated with the highest proportion of fatal cases (6% of its reported AEs). Cardiovascular AEs represented a significant portion of AEs for phentermine (31%) and liraglutide (23%) [25].

Although semaglutide and liraglutide show antidepressant and anxiolytic effects in animal models of depression and anxiety, in July 2023, the European Medicine Agency (EMA)’s safety committee, the Pharmacovigilance Risk Assessment Committee (PRAC), was reviewing data on the risk of thoughts of suicide and self-harm associated with GLP-1 receptor agonists including semaglutide (Ozempic) and liraglutide (Saxenda) [26]. Therefore, these medications should be used cautiously and closely monitored in patients with a history or risk of psychiatric disorders [26]. Furthermore, as previously mentioned, there is a lack of data on psychiatric adverse events associated with GLP-1 receptor agonists reported in clinical trials [13–15].

### Aim

The aim of this study was to identify and analyse the reported psychiatric adverse events and clinical outcomes for three anti-diabetic and anti-obesity medications, namely semaglutide, liraglutide, and tirzepatide based on spontaneous reports from the EudraVigilance database.

### Ethics approval

Data derived from the EudraVigilance database are anonymous and publicly available, therefore, no access authorization was required. The access policy of European Medicines Agency (EMA) states that "No authorization for accessing the ICSR (Level 1) data set by means of the adrreports.eu portal is required i.e., all academic researchers can access adverse reaction data of interest”

## Method

The EudraVigilance is a centralized EU database maintained by the EMA and supports monitoring the safe and effective use of medicines that have been authorized or studied in clinical trials in the EU. A medication safety database was previously used to report adverse effects with pharmaceuticals [27, 28]. All individual case safety reports (ICSR) for semaglutide, liraglutide, and tirzepatide reported to the EudraVigilance database from 01/01/2021 to 30/05/2023 were analysed for psychiatric adverse events.

Liraglutide Saxenda® was the first anti-obesity drug approved by the EMA in January 2015, followed by semaglutide Wegovy® in January 2022. Tirzepatide Mounjaro®, a new Dual-Targeted Treatment for T2DM, was approved in May 2022 and recently approved for weight management. The analysis covered the period of approval of these drugs and compared the adverse events in specific time intervals.

Line listing search was conducted for data collection. The search included all spontaneous reports of psychiatric adverse drug reactions, regardless of the dose, frequency, or route of administration of the selected medication. The following information was recorded for each ICSR; a unique identifier number, age group (age groups: ≥ 85 years old, 65–84, 18–64 and 12–17 years, non-specified age), sex, date of reporting, origin of the report (EU or non-EU), reporter’s profession (healthcare professional or non-healthcare professional), concomitant conditions and psychotic ADR outcomes and seriousness. Each ICSR may include more than one suspected ADR.

The results of the line listing search were then exported to a Microsoft Excel file and data were cleaned by removing irrelevant columns and fields, tabulated and presented according to the type of psychiatric ADR and clinical outcome, by gender and age. The clinical outcomes were categorised into four sections: (i) recovered/resolved, (ii) recovering/resolving, (iii) not recovered/not resolved, (iv) fatal/life-threatening, and (v) unknown outcome.

Descriptive statistics were used to describe the study population characteristics. Variables were reported as absolute numbers and percentages. Number of reports is the denominator for all proportions unless indicated otherwise. All analyses were performed using IBM SPSS Statistics for Windows version 20.0 (IBM Corp., Armonk, NY, USA).

## Results

**Fig. 1:**
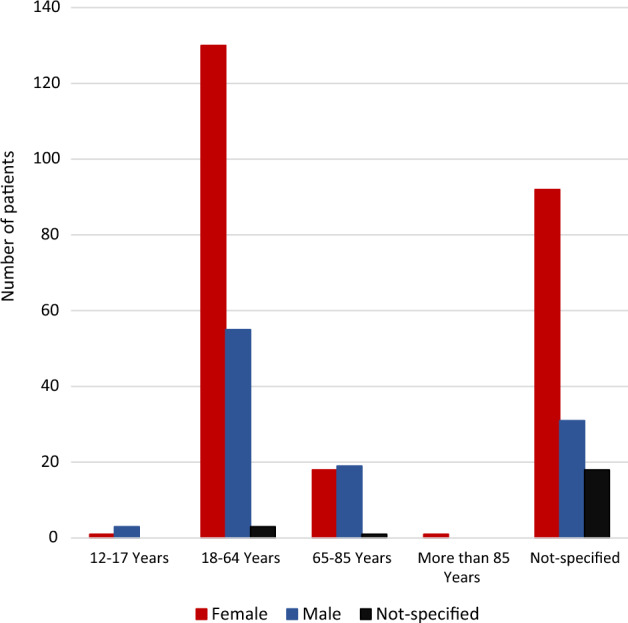
Age and sex distribution of patients with psychiatric adverse events associated with semaglutide, liraglutide and tirzepatide

During the study period, 31,444 ADR reports were identified: semaglutide (n = 13,956; 44.4%), liraglutide (n = 16,748; 53.3%), and tirzepatide (n = 740; 2.3%). There were 372 reports with psychiatric adverse events submitted to the EudraVigilance database during the study period (372/31,444; 1.18%) and 480 adverse events in total, as each report may contain more than one ADR. Women accounted for 65% (n = 242) of the reports whereas men accounted for 29% (n = 108). The sex in the remaining 6% (n = 22) was not specified. Figure 1 illustrates the age and sex distribution of the patients with reported psychiatric adverse events.

Depression was the most commonly reported adverse event of the total reports (n = 187; 50.3%), followed by anxiety (n = 144; 38.7%) and suicidal ideation (n = 73; 19.6%) in Table 1. Nine deaths were reported (8 for liraglutide; 1 for semaglutide), and 11 life-threatening outcomes (4 for liraglutide; 7 for semaglutide) were reported during the study period. The fatal outcomes occurred mainly among men (8 out of 9) due to completed suicidal attempts and depression in Tables 2 and 3 respectively.

**Table 1:** General characteristics of patients with psychiatric adverse events

Total	Liraglutide		Semaglutide		Tirzepatide		Total	
	147		210		15		372	
	N	%	N	%	N	%	N	%
*Report year*								
2021	43	29.3	63	30.0	0	0.0	106	28.5
2022	70	47.6	81	38.6	5	33.3	156	41.9
2023	34	23.1	66	31.4	10	66.7	110	29.6
*Age group in years*								
12–17	4	2.7	0	0.0	0	0.0	4	1.1
18–64	74	50.3	103	49.0	11	73.3	188	50.5
65–85	7	4.8	30	14.3	1	6.7	38	10.2
More than 85	0	0.0	1	0.5	0	0.0	1	0.3
Not specified	62	42.2	76	36.2	3	20.0	141	37.9
*Sex*								
Male	113	76.9	121	57.6	8	53.3	108	29.0
Female	33	22.4	72	34.3	3	20.0	242	65.1
Not specified	1	0.7	17	8.1	4	26.7	22	5.9
*Country of report*								
European Economic Area	50	34.0	72	34.3	0	0	122	32.8
Non-European Economic Area	97	66.0	138	65.7	15	100	250	67.2
*The reporter*								
Healthcare Professional	78	53.1	108	51.4	7	46.7	193	51.9
Non-Healthcare Professional	69	46.9	102	48.6	8	53.3	179	48.1
*Psychiatric adverse events*^*^*^								
Anxiety	60	40.8	71	33.8	13	86.7	144	38.7
Depression	66	44.9	117	55.7	4	26.7	187	50.3
Suicide attempt	7	6.1	5	2.4	0	0.0	12	4.6
Suicidal ideation	29	19.4	40	19.0	4	26.7	73	19.6
Completed suicide	4	0.0	0	1.9	0	0.0	4	1.1
Depression with suicidal	4	4.1	6	1.9	0	0.0	10	2.7
Suspected suicide	2	1.4	0	0.0	0	0.0	2	0.5
Other*	11	7.5	34	16.2	0	0.0	45	12.1

^^^The total number of adverse events is higher than the number of patients/reports as a single report may contain more than one adverse event

*Includes affective disorders, Hallucinations, Mania, Mood swings and one case of suicidal behavior with liraglutide

**Table 2:** Outcome and severity of psychiatric adverse events reported in the EudraVigilance database by age group and sex

Sex	Age	Not recovering/not resolving		Recovered/resolved		Recovering/resolving		Life-threatening/fatal		Unknown	
		N	%	N	%	N	%	N	%	N	%
Male	12–17	0	0	0	0	0	0	1	5.0	2	1.2
	18–64	11	16.7	10	11.4	6	17.6	8	40.0	20	12.2
	65–85	4	6.1	6	6.8	2	5.9	0	0.0	7	4.3
	More than 85	0	0.0	0	0.0	0	0.0	0	0.0	0	0.0
	Not specified	3	4.5	7	8.0	4	11.8	3	15.0	14	8.5
	**Total**	**18**	**27.3**	**23**	**26.1**	**12**	**35.3**	**12**	**60.0**	**43**	**26.2**
Female	12–17	0	0.0	0	0.0	0	0.0	0	0.0	1	0.6
	18–64	23	34.8	31	35.2	15	44.1	3	15.0	58	35.4
	65–85	5	7.6	5	5.7	1	2.9	0	0.0	7	4.3
	More than 85	0	0.0	0	0.0	0	0.0	1	5.0	0	0.0
	Not specified	16	24.2	24	27.3	6	17.6	4	20.0	42	25.6
	**Total**	**44**	**66.7**	**60**	**68.2**	**22**	**64.7**	**8**	**40.0**	**108**	**65.9**
Not specified	12–17	0	0.0	0	0.0	0	0.0	0	0.0	0	0.0
	18–64	0	0.0	1	1.1	0	0.0	0	0.0	2	1.2
	65–85	0	0.0	0	0.0	0	0.0	0	0.0	1	0.6
	More than 85	0	0.0	0	0.0	0	0.0	0	0.0	0	0.0
	Not specified	4	6.1	4	4.5	0	0.0	0	0.0	10	6.1
	**Total**	**4**	**6.1**	**5**	**5.7**	**0**	**17.6**	**0**	**40.0**	**13**	**7.9**
**Grand total**		**66**		**88**		**34**		**20**		**164**	

*Percentage of the total number of reports in the respective outcome group

**Table 3:** Details of the fatal and life-threatening outcomes

No.	Outcome	Age in years	Sex	Adverse event-outcome	Medication list
1	Death	18–64	Male	Completed suicide-Death	Liraglutide + Metformin + Perindopril
2		18–64	Male	Completed suicide-Death	Liraglutide + Metformin + Perindopril
3		18–64	Male	Completed suicide-Death	Liraglutide + Rosuvastatin
4		18–64	Male	Completed suicide-Death	Liraglutide + Metformin + Perindopril
5		18–64	Male	Suspected suicide-Death	Liraglutide + Insulin Glargine + Paracetamol + Lisinopril
6		18–64	Male	Suspected suicide-Death	Liraglutide + Insulin Glargine + Paracetamol + Lisinopril
7		Not specified	Male	Depression*-Death	Liraglutide + Sitagliptin
8		Not specified	Male	Depression**-Death	Liraglutide + Sitagliptin + Metformin
9		More than 85	Female	Depression-Death	Semaglutide
10	Life-threatening condition	12–17	Male	Suicide attempt-Recovering	Liraglutide
11		18–64	Male	Depression, suicidal-Unknown	Liraglutide
12		18–64	Male	Delerium^-Unknown	Semaglutide + Ramipril + Chlorhexidine + Metformin
13		Not specified	Male	Anxiety-Not Recovered	Semaglutide
14		18–64	Female	Suicidal ideation-Recovering	Liraglutide
15		18–64	Female	Suicidal ideation-Recovered	Semaglutide
16		18–64	Female	Depression-Recovering	Semaglutide
17		Not specified	Female	Suicidal ideation, aggravated depression-Unknown	Liraglutide + Fluoxetine Hydrochloride
18		Not specified	Female	Suicidal ideation-Recovering	Semaglutide
19		Not specified	Female	Suicidal ideation-Not Recovered	Semaglutide
20		Not specified	Female	Suicide attempt-Unknown	Semaglutide

*The patient had pancreatic cancer

**The patient had cancer of the prostate

^The patient had cerebral haemorrhage and acute kidney injury

### Suicidal adverse events

The database recorded 102 adverse events related to suicide. The events included suicidal ideations (n = 73), suicidal attempts (n = 12), depression with suicidal ideations (n = 10), completed suicides (death) (n = 4), suspected suicides (n = 2) and suicidal behavior (n = 1). Overall, in terms of total reported suicidal events, 50.0% occurred with semaglutide (51/102; 50%) and constituted 24.3% of the total semaglutide psychiatric adverse events reports (51/210; 24.3%). For liraglutide, out of the total reported suicidal events, 47% occurred with liraglutide (48/102; 47%) and constituted 32.7% of the total liraglutide psychiatric adverse events reported (48/147; 32.7%). Four of the total reported suicidal events, (4/102; 3.9%), were associated with tirzepatide which also constituted 26.7% of the total tirzepatide psychiatric adverse events (4/15; 26.7%). Sixty-two percent of suicidal events occurred in females and almost half of the reports (44.6%) occurred in the 18–64 age group. Table 4 (Electronic Supplementary Material) illustrates the age and sex distribution and the outcome of the suicide-related adverse events.

## Discussion

### Statement of key findings

Between January 2021 and May 2023, there were 372 adverse event reports with 481 psychiatric events associated with three GLP-1 agonists (semaglutide, liraglutide, tirzepatide) reported in the EMA EudraVigilance database. Some of the events resulted in severe consequences including suicidal attempts and completed suicides (deaths). The number of reported events for these medicines increased over the study years, which may be due to their increased use. There were a lower number of reports for tirzepetide, likely because it was more recently approved by the FDA, in 2022, for the treatment of T2DM.

Approximately 50% of the reports occurred in the 18–64-year age group and 65% of the reports were from females. This may be due to the higher use of these medications in adult women. A previous database study conducted using the Clinical Practice Research Datalink in the UK found that among 589 patients prescribed a GLP-1 receptor agonist, 56.4% were female and the median age was 54 years.

A comparison between the three studied medications revealed more fatal adverse events associated with liraglutide (8 patients had a fatal outcome out of 147 ICSR) compared with semaglutide (one death out of 210 ICSR) and none with tirzepatide (out of 15 ICSR). Fatal outcomes were primarily the result of completed suicide and were more common in males in the 18–64 age group [29]. In this study, half (50.0%) of the reported suicidal events occurred with semaglutide followed by liraglutide, whereas only four occurred with tirzepatide.

A pooled post-hoc analysis of neuropsychiatric safety data across weight management trials with liraglutide revealed a “small numerical imbalance” in suicidal ideation with liraglutide compared with placebo. Nine (0.3%) patients in the liraglutide group vs 2 (0.1%) individuals in the placebo group reported suicidal ideation or behavioral adverse events [30]. Suicidal ideation events were detected for 8 of 11 individuals using the Columbia-Suicide Severity Rating Scale (C-SSRS), all of which occurred in the liraglutide group [30].

In a study of 201 adolescents with obesity, it was found that a smaller percentage of participants in the semaglutide group reported psychiatric adverse events compared to the placebo group (7% vs. 15%). However, the study did not provide any details on the type of symptoms or severity experienced by the participants [31].

Of note, there is evidence to suggest a link between obesity, being overweight, and psychiatric events such as depression, anxiety and suicidal ideation [32–34]. The relationship between obesity and psychiatric events is complex and can be bi-directional. Although obesity may increase the risk of psychiatric disorders, mental health conditions also contribute to the development or worsening of obesity through various mechanisms such as emotional eating and decreased motivation for physical activity [34, 35].

The relationship between obesity and depression has been extensively studied in scientific literature with some studies showing significant associations while others yielding contradictory results. A large proportion of cross-sectional studies are heterogeneous in their design making it difficult to draw clear conclusions [35, 36].

In the US FDA’s FAERS, 1200 reports of adverse reactions had been reported for semaglutide since 2018, including 60 cases of suicidal ideation and 7 suicide attempts, (5%, 0.5%) respectively. For liraglutide, of > 35,000 reports of adverse reactions, there have been 71 cases of suicidal ideation, 28 suicide attempts and 25 completed suicides, (2%, 0.8%, 0.7%) respectively [37].

While this study presents one perspective on liraglutide's safety profile, data from the US FAERS database (January 2013–June 2020) revealed 82 reported cases of anxiety (11%), 376 life-threatening events (9%, cause unclear), and 217 fatalities (5%) associated with liraglutide use [25].

## Strengths and weaknesses

To the best of the authors’ knowledge, this is the first study that evaluated in a real-world setting the psychiatric adverse events associated with three anti-diabetic and anti-obesity GLP1 receptor agonist medications. Although a low number of psychiatric adverse events were reported in this study, 20 patients had a fatal or life-threatening outcome among the severe clinical outcomes associated with the three drugs. However, this finding should be interpreted with caution because of the nature of the data and the passive reporting system used. Other limitations include the use of pharmacovigilance databases. Data from the EudraVigilance and other drug safety databases relies on spontaneous reporting which may introduce reporting bias, underreporting adverse events and missing information. Furthermore, the observational design of the study contributes to the lack of detailed information on underlying patient conditions and hinders the establishment of a causal relationship between GLP-1 receptor agonists and adverse psychiatric effects. The lack of a denominator for the total population taking the medications, where reports came from European and non-European countries, makes it difficult to determine if there is a significant risk.

## Interpretation and further research

Psychiatric adverse events appear to be a potential concern with semaglutide, liraglutide, and tirzepatide but their true risk is unknown. Therefore, healthcare professionals should be aware of the potential for psychiatric adverse events, particularly depression, anxiety and suicidal ideation associated with semaglutide, liraglutide, and tirzepatide [38]. Patients should be encouraged to report any changes in mood or behavior to their healthcare provider and drug safety authority [25].

There are a variety of interventions to assess a patient's risk of suicide, depending on their individual situation. Screening tools, such as the Patient Health Questionnaire-9 (PHQ-9) and the Columbia-Suicide Severity Rating Scale (C-SSRS), can be used to identify individuals at risk of suicide. These tools are easy to administer and can be incorporated into electronic medical records [39, 40].

The decision to start drug therapy for obesity should be made on a case-by-case basis, after weighing the risks and benefits. It is important to set clear treatment goals and to consider the patient's preferences and tolerability as these can have an impact on adherence [38].

In the future, it is recommended to include a more significant number of follow-up studies based on unified criteria to understand better the relationship between obesity and psychiatric disorders, including suicide, anxiety and depression [36, 37]. Furthermore, further research is needed to identify the psychiatric adverse events associated with new anti-obesity medications. Additional well-conducted and well-reported studies are needed, accompanied by the use of accepted definitions and causality assessment tools to address the lack of published literature regarding this issue.

## Conclusion

Although adverse psychiatric events account for only 1.2% of the total reports associated with semaglutide, liraglutide, and tirzepatide, 20 patients had a fatal or life-threatening outcome. The seriousness of these adverse events warrants additional research to explore the causal relationship. Neuropsychiatric safety should be a key consideration in any clinical risk–benefit assessment associated with GLP1 anti-obesity medications and the analysis provided here contribute to thoughtful and objective decision-making and dialogue between patients and clinicians.

### Supplementary Information

Below is the link to the electronic supplementary material.

Supplementary file1 (DOCX 24 kb)
