# Supplementary material for: Psychiatric adverse events associated with semaglutide, liraglutide and tirzepatide: a pharmacovigilance analysis of individual case safety reports submitted to the EudraVigilance database
Source: Int J Clin Pharm. 2024 Jan 24;46(2):488–95. doi: 10.1007/s11096-023-01694-7 (PMC10960895; doi:10.1007/s11096-023-01694-7)
Supplement: Supplementary file 1 — Supplementary file1 (DOCX 24 kb) [file 11096_2023_1694_MOESM1_ESM.docx]

**Electronic supplementary**

Table 4: Outcome and details of spontaneous report with suicidal events associated related to semaglutide (A), liraglutide (B), and tirzepatide (C).

**A: Details of spontaneous report with suicidal events associated related to liraglutide.**

| **Receipt Date** | **The reporter** | **Source Country** | **Age group in years** | **Sex** | **Adverse Event (Outcome)** |
| --- | --- | --- | --- | --- | --- |
| 11/01/2021 | Non Healthcare Professional | Non-EU | N/S | Female | Suicidal ideation (Not Recovered/Not Resolved) |
| 26/03/2021 | Healthcare Professional | Non-EU | 65-85 | Female | Suicidal ideation (Unknown) |
| 20/05/2021 | Healthcare Professional | Non-EU | N/S | Male | Suicidal ideation, Depression (Unknown ), |
| 27/05/2021 | Healthcare Professional | Non-EU | 18-64 | Male | Completed suicide (Fatal - Results in Death) |
| 02/06/2021 | Healthcare Professional | Non-EU | N/S | Female | Suicidal ideation (Recovered/Resolved) |
| 03/06/2021 | Healthcare Professional | Non-EU | N/S | Female | Suicidal ideation, Depression (Recovered/Resolved) |
| 11/06/2021 | Healthcare Professional | Non-EU | N/S | Female | Suicide attempt (Unknown) |
| 02/08/2021 | Healthcare Professional | Non-EU | N/S | Female | Suicidal ideation (Recovered/Resolved) |
| 02/09/2021 | Non Healthcare Professional | Non-EU | 65-85 | Female | Suicidal ideation (Recovering/Resolving) |
| 21/10/2021 | Healthcare Professional | Non-EU | 18-64 | Male | Completed suicide (Fatal - Results in Death) |
| 03/11/2021 | Healthcare Professional | Non-EU | 18-64 | Male | Completed suicide (Fatal - Results in Death) |
| 24/01/2022 | Non Healthcare Professional | Non-EU | N/S | Female | Depression suicidal (Not Recovered/Not Resolved) |
| 24/01/2022 | Non Healthcare Professional | Non-EU | N/S | Female | Suicidal ideation (Not Recovered/Not Resolved) |
| 11/04/2022 | Healthcare Professional | Non-EU | 18-64 | Male | Suicidal ideation (Unknown) |
| 11/05/2022 | Non Healthcare Professional | Non-EU | N/S | Female | Depression suicidal (Unknown) |
| 20/05/2022 | Healthcare Professional | Non-EU | 18-64 | Female | Suicidal ideation (Recovered/Resolved) |
| 10/06/2022 | Healthcare Professional | Non-EU | 18-64 | Male | Completed suicide (Fatal - Results in Death) |
| 27/06/2022 | Healthcare Professional | EU | N/S | Female | Suicidal ideation (Recovered/Resolved) |
| 26/07/2022 | Healthcare Professional | Non-EU | 18-64 | Male | Depression suicidal (Life Threatening) |
| 28/07/2022 | Healthcare Professional | Non-EU | N/S | Female | Suicidal behaviour (Recovered/Resolved) |
| 17/08/2022 | Healthcare Professional | Non-EU | N/S | Female | Suicidal ideation (Unknown) |
| 08/09/2022 | Non Healthcare Professional | Non-EU | N/S | Female | Suicidal ideation (Unknown) |
| 09/09/2022 | Healthcare Professional | Non-EU | 18-64 | Male | Suicidal ideation (Unknown) |
| 22/09/2022 | Non Healthcare Professional | Non-EU | 18-64 | Female | Suicidal ideation (Recovered/Resolved) |
| 13/10/2022 | Non Healthcare Professional | EU | 18-64 | Female | Suicidal ideation (Not Recovered/Not Resolved) |
| 28/10/2022 | Non Healthcare Professional | EU | N/S | Female | Suicidal ideation (Unknown) |
| 03/11/2022 | Healthcare Professional | EU | 18-64 | Female | Suicidal ideation (Recovered/Resolved) |
| 16/11/2022 | Non Healthcare Professional | Non-EU | 12-17 | Male | Suicide attempt (Recovering/Resolving - Life Threatening) |
| 12/12/2022 | Healthcare Professional | Non-EU | N/S | Female | Depression (Recovered/Resolved), Suicidal ideation (Unknown) |
| 28/12/2022 | Healthcare Professional | Non-EU | 18-64 | Male | Suicide attempt (Unknown - Prolonged Hospitalisation) |
| 09/01/2023 | Healthcare Professional | Non-EU | N/S | Male | Suicidal ideation (Recovering/Resolving) |
| 16/01/2023 | Healthcare Professional | Non-EU | 18-64 | Male | Suspected suicide (Fatal - Results in Death) |
| 16/01/2023 | Healthcare Professional | Non-EU | 18-64 | Male | Suspected suicide (Fatal - Results in Death) |
| 31/01/2023 | Non Healthcare Professional | EU | 18-64 | Female | Suicidal ideation (Unknown) |
| 10/02/2023 | Healthcare Professional | Non-EU | 65-85 | Female | Suicide attempt (Recovered/Resolved - Prolonged Hospitalisation) |
| 01/03/2023 | Non Healthcare Professional | Non-EU | 18-64 | Female | Suicidal ideation (Not Recovered/Not Resolved - Disabling) |
| 14/03/2023 | Non Healthcare Professional | Non-EU | 18-64 | Female | Suicidal ideation (Recovered/Resolved) |
| 29/03/2023 | Non Healthcare Professional | Non-EU | 18-64 | Male | Suicide attempt (Unknown) |
| 31/03/2023 | Healthcare Professional | Non-EU | 12-17 | Male | Depression suicidal (Unknown - Prolonged Hospitalisation) |
| 07/04/2023 | Non Healthcare Professional | Non-EU | N/S | Female | Suicidal ideation (Unknown - Life Threatening) |
| 10/04/2023 | Non Healthcare Professional | Non-EU | 18-64 | Female | Suicidal ideation (Recovering/Resolving) |
| 17/04/2023 | Non Healthcare Professional | Non-EU | 12-17 | Female | Suicidal ideation (Recovered/Resolved) |
| 05/05/2023 | Healthcare Professional | Non-EU | N/S | Male | Suicide attempt (Unknown) |
| 08/05/2023 | Healthcare Professional | Non-EU | N/S | Female | Suicidal ideation (Unknown) |
| 16/05/2023 | Healthcare Professional | Non-EU | 12-17 | Male | Suicidal ideation (Not Recovered/Not Resolved) |
| 24/05/2023 | Healthcare Professional | Non-EU | 65-85 | Female | Suicide attempt (Recovered/Resolved - Prolonged Hospitalisation), |
| 26/05/2023 | Healthcare Professional | EU | 18-64 | Female | Suicidal ideation (Recovered/Resolved) |

N/S; Not Specified

**B: Details of spontaneous report with suicidal events associated related to semaglutide.**

| **Receipt Date** | **The reporter** | **Source Country** | **Age group in years** | **Sex** | **Adverse event (Outcome)** |
| --- | --- | --- | --- | --- | --- |
| 16/12/2021 | Non Healthcare Professional | EU | 18-64 | Female | Suicidal ideation (6.5d - Recovered/Resolved) |
| 03/12/2021 | Healthcare Professional | EU | N/S | Female | Suicide attempt (Unknown - Prolonged Hospitalisation) |
| 21/10/2021 | Healthcare Professional | Non EU | 18-64 | Female | Suicidal ideation (Recovered/Resolved) |
| 14/10/2021 | Non Healthcare Professional | Non EU | N/S | Male | Depression suicidal (Unknown) |
| 28/09/2021 | Healthcare Professional | Non EU | 65-85 | Male | Suicidal ideation (Recovered/Resolved with Sequelae - Disabling) |
| 14/09/2021 | Non Healthcare Professional | Non EU | 18-64 | Female | Depression suicidal (Recovering/Resolving) |
| 21/07/2021 | Non Healthcare Professional | Non EU | N/S | Female | Suicidal ideation (Recovered/Resolved) |
| 14/05/2021 | Non Healthcare Professional | Non EU | 65-85 | Male | Suicidal ideation (Unknown) |
| 29/03/2021 | Non Healthcare Professional | Non EU | N/S | Female | Suicidal ideation (Recovered/Resolved) |
| 19/02/2021 | Healthcare Professional | Non EU | 18-64 | Female | Suicidal ideation (Unknown) |
| 29/12/2022 | Healthcare Professional | EU | 18-64 | Female | Suicidal ideation (Recovering/Resolving - Prolonged Hospitalisation) |
| 22/11/2022 | Healthcare Professional | Non EU | 18-64 | Female | Suicidal ideation (Unknown) |
| 18/11/2022 | Healthcare Professional | Non EU | 18-64 | Female | Suicidal ideation (Unknown) |
| 03/11/2022 | Healthcare Professional | Non EU | N/S | Female | Suicidal ideation (Not Recovered/Not Resolved) |
| 21/10/2022 | Healthcare Professional | Non EU | N/S | Female | Suicidal ideation (Recovered/Resolved) |
| 06/09/2022 | Non Healthcare Professional | Non EU | N/S | Female | Depression (Unknown), Suicidal ideation (Unknown) |
| 22/06/2022 | Non Healthcare Professional | Non EU | 18-64 | Female | Depression suicidal (Not Recovered/Not Resolved) |
| 26/04/2022 | Non Healthcare Professional | Non EU | 18-64 | Male | Suicidal ideation (Unknown) |
| 01/03/2022 | Healthcare Professional | Non EU | N/S | Female | Depression suicidal (Recovered/Resolved) |
| 17/05/2023 | Healthcare Professional | Non EU | 18-64 | Male | Suicidal ideation (Recovered/Resolved - Prolonged Hospitalisation) |
| 12/05/2023 | Healthcare Professional | Non EU | N/S | Female | Suicidal ideation (Recovering/Resolving) |
| 13/04/2023 | Healthcare Professional | EU | 18-64 | Female | Depression suicidal (Recovered/Resolved) |
| 31/03/2023 | Healthcare Professional | Non EU | N/S | Female | Suicide attempt (Unknown - Prolonged Hospitalisation) |
| 07/03/2023 | Healthcare Professional | EU | 18-64 | Female | Suicidal ideation (Not Recovered/Not Resolved) |
| 20/02/2023 | Non Healthcare Professional | Non EU | N/S | Female | Suicidal ideation (Recovered/Resolved) |
| 20/02/2023 | Healthcare Professional | Non EU | N/S | N/S | Suicidal ideation (Unknown) |
| 10/02/2023 | Healthcare Professional | Non EU | 18-64 | Male | Suicidal ideation (2d - Recovered/Resolved) |
| 10/02/2023 | Healthcare Professional | Non EU | 18-64 | Male | Suicidal ideation (Recovering/Resolving) |
| 03/02/2023 | Healthcare Professional | Non EU | 18-64 | Female | Depression suicidal (32d - Recovered/Resolved) |
| 28/09/2021 | Non Healthcare Professional | Non EU | N/S | Female | Suicidal ideation (Recovered/Resolved) |
| 23/07/2021 | Healthcare Professional | Non EU | 18-64 | Female | Suicidal ideation (Not Recovered/Not Resolved) |
| 14/12/2022 | Non Healthcare Professional | Non EU | 18-64 | Female | Suicidal ideation (Unknown) |
| 24/08/2022 | Healthcare Professional | Non EU | 18-64 | Female | Suicidal ideation (Recovered/Resolved) |
| 18/08/2022 | Non Healthcare Professional | Non EU | 18-64 | Male | Suicidal ideation (Recovered/Resolved) |
| 18/08/2022 | Healthcare Professional | Non EU | N/S | Female | Suicidal ideation (Recovered/Resolved) |
| 01/08/2022 | Healthcare Professional | Non EU | N/S | Female | Suicide attempt (Unknown) |
| 24/06/2022 | Non Healthcare Professional | EU | 18-64 | Female | Suicidal ideation (Recovered/Resolved - Life Threatening) |
| 27/04/2022 | Non Healthcare Professional | Non EU | N/S | Female | Suicide attempt (Unknown - Life Threatening) |
| 18/04/2022 | Healthcare Professional | Non EU | 18-64 | Male | Suicidal ideation (Not Recovered/Not Resolved |
| 24/05/2023 | Non Healthcare Professional | Non EU | N/S | Female | Suicidal ideation (Recovering/Resolving - Life Threatening) |
| 22/05/2023 | Non Healthcare Professional | Non EU | N/S | N/S | Suicidal ideation (Unknown) |
| 17/05/2023 | Healthcare Professional | Non EU | 18-64 | Male | Suicide attempt (Recovered/Resolved -Prolonged Hospitalisation) |
| 16/05/2023 | Non Healthcare Professional | Non EU | 18-64 | Male | Suicidal ideation (Not Recovered/Not Resolved) |
| 16/05/2023 | Healthcare Professional | Non EU | N/S | Female | Suicidal ideation (Not Recovered/Not Resolved - Life Threatening) |
| 27/04/2023 | Healthcare Professional | Non EU | 18-64 | Female | Suicidal ideation (Recovered/Resolved) |
| 05/04/2023 | Non Healthcare Professional | Non EU | N/S | Male | Suicidal ideation (Recovered/Resolved) |
| 17/03/2023 | Healthcare Professional | Non EU | N/S | N/S | Suicidal ideation (Not Recovered/Not Resolved) |
| 17/03/2023 | Healthcare Professional | Non EU | N/S | N/S | Suicidal ideation (Recovered/Resolved) |
| 17/03/2023 | Healthcare Professional | Non EU | N/S | N/S | Suicidal ideation (Recovered/Resolved) |
| 16/03/2023 | Healthcare Professional | Non EU | N/S | N/S | Suicidal ideation (Not Recovered/Not Resolved) |
| 07/02/2023 | Non Healthcare Professional | Non EU | N/S | N/S | Suicidal ideation (Unknown) |

N/S; Not Specified

**C: Details of spontaneous report with suicidal events associated related to tirzepatide**

| **Receipt Date** | **The reporter** | **Source Country** | **Age group in years** | **Sex** | **Adverse Event (Outcome)** |
| --- | --- | --- | --- | --- | --- |
| 03/05/2023 | Non Healthcare Professional | Non EU | 18-64 | Male | Suicidal ideation (Not Recovered/Not Resolved) |
| 11/04/2023 | Non Healthcare Professional | Non EU | N/S | N/S | Suicidal ideation (Unknown) |
| 09/12/2022 | Non Healthcare Professional | Non EU | 18-64 | Female | Suicidal ideation (Not Recovered/Not Resolved) |
| 25/10/2022 | Healthcare Professional | Non EU | 65-85 | Male | Suicidal ideation (Not Recovered/Not Resolved) |

N/S; Not Specified
